# Lack of Protection following Passive Transfer of Polyclonal Highly Functional Low-Dose Non-Neutralizing Antibodies

**DOI:** 10.1371/journal.pone.0097229

**Published:** 2014-05-12

**Authors:** Anne-Sophie Dugast, Ying Chan, Michelle Hoffner, Anna Licht, Joseph Nkolola, Hualin Li, Hendrik Streeck, Todd J. Suscovich, Musie Ghebremichael, Margaret E. Ackerman, Dan H. Barouch, Galit Alter

**Affiliations:** 1 Ragon Institute of Massachusetts General Hospital, Harvard University and Massachusetts Institute of Technology, Cambridge, Massachusetts, United States of America; 2 Center for Virology and Vaccine Research, Beth Israel Deaconess Medical Center, Boston, Massachusetts, United States of America; 3 Thayer School of Engineering, Dartmouth College, Hanover, New Hampshire, United States of America; 4 Military HIV Research Program, Henry Jackson Foundation, Rockville, Maryland, United States of America; University of Massachusetts Medical Center, United States of America

## Abstract

Recent immune correlates analysis from the RV144 vaccine trial has renewed interest in the role of non-neutralizing antibodies in mediating protection from infection. While neutralizing antibodies have proven difficult to induce through vaccination, extra-neutralizing antibodies, such as those that mediate antibody-dependent cellular cytotoxicity (ADCC), are associated with long-term control of infection. However, while several non-neutralizing monoclonal antibodies have been tested for their protective efficacy *in vivo*, no studies to date have tested the protective activity of naturally produced polyclonal antibodies from individuals harboring potent ADCC activity. Because ADCC-inducing antibodies are highly enriched in elite controllers (EC), we passively transferred highly functional non-neutralizing polyclonal antibodies, purified from an EC, to assess the potential impact of polyclonal non-neutralizing antibodies on a stringent SHIV-SF162P3 challenge in rhesus monkeys. Passive transfer of a low-dose of ADCC inducing antibodies did not protect from infection following SHIV-SF162P3 challenge. Passively administered antibody titers and gp120-specific, but not gp41-specific, ADCC and antibody induced phagocytosis (ADCP) were detected in the majority of the monkeys, but did not correlate with post infection viral control. Thus these data raise the possibility that gp120-specific ADCC activity alone may not be sufficient to control viremia post infection but that other specificities or Fc-effector profiles, alone or in combination, may have an impact on viral control and should be tested in future passive transfer experiments.

## Introduction

While a large number of novel, broadly neutralizing antibodies have been described recently, the induction of such antibodies through vaccination in humans has proven difficult [1]. Neutralizing antibodies develop in approximately one third of infected patients, only after two to three years of infection [2]–[4]. However, the mechanism by which these individuals are able to induce broad humoral immunity, though often after viral escape, is not understood.

Yet beyond neutralization, antibodies possess a large variety of additional extra-neutralizing properties that allow them to control and eliminate infections, including antibody-dependent cellular cytotoxicity (ADCC), antibody-dependent cellular phagocytosis (ADCP) of immune complexes, antibody-dependent cellular viral inhibition (ADCVI) [5] and complement activation [6], [7]. Antibodies mediate these functions via the recruitment of the innate immune system through Fc/Fc-γ receptor (FcγR) interactions or the recruitment of complement. Moreover the role of these extra-neutralizing functions in the control of HIV have been most clearly illustrated using a variant of b12 with abrogated capacity to interact with Fc receptors that reduced the protective efficacy of passively transferred b12 in rhesus macaques [8].

Non-neutralizing antibodies with innate immune–stimulating activities (e.g., ADCC-inducing antibodies) have been shown to play a crucial role in the clearance of several viral infections [9]–[11]. In HIV [12]–[16], antibodies with innate immune–recruiting activity have been detected in acutely infected individuals [12] and subjects who spontaneously control HIV infection in the absence of therapy [12], [17]. Furthermore, strong ADCVI responses are associated with slower disease progression in long-term non-progressors [12], [13], [18]–[20], suggesting that antibodies with specific innate immune–activating properties may contribute to viral containment *in vivo*. Additionally, vaccine-elicited, non-neutralizing-ADCC-inducing antibodies [21]–[24] or the passive transfer of ADCC-inducing-non-neutralizing antibodies [25] have been repeatedly shown to contribute to antiviral control post-infection, further highlighting the importance of non-neutralizing antibody effector functions in the control of HIV. Most recently, the immune correlates analysis of the RV144 vaccine trial demonstrated that ADCC activity, in the absence of vaccine-elicited IgA responses, was associated with a reduced risk of infection among vaccinees, supporting a role for vaccine-elicited non-neutralizing antibodies in protection for the first time [26].

Importantly, unlike neutralizing antibodies that target a limited network of neutralizing epitopes on the viral envelope, non-neutralizing antibodies may require the synergistic targeting of a much larger spectrum of epitopes to form an immune complex. Together, this targeting provides the avidity necessary to trigger the relatively low-affinity innate immune receptors, thereby allowing for rapid target cell elimination. Therefore, while non-neutralizing monoclonal antibodies, or combinations of several monoclonal antibodies, have afforded only limited protection from infection, it is plausible that a polyclonal pool of highly functional antibodies that target a broad range of epitopes may increase the ability of non-neutralizing antibodies to prevent or control HIV infection. Here we assessed whether polyclonal antibodies from a patient with robust non-neutralizing activity could provide protection from infection or mediate virological control following SHIV-SF162P3 challenge in rhesus macaques. Overall, passive transfer of a low-dose of a polyclonal pool of ADCC-inducing non-neutralizing antibodies did not provide sterilizing protection following intrarectal challenge with SHIV-SF162P3. Ancillary analyzes demonstrate the selective persistence of IgG1 Abs and functional responses against gp120, but not gp41 after transfer in the blood, but not at the mucosa, potentially accounting for lack of protection from challenge.

## Materials and Methods

### Patients

Samples from a total of 15 elite controllers were included in this study, and IgG from one elite controller with robust innate–recruiting activity in the absence of neutralization was selected for passive transfer. In addition, a pool of IgG derived from five uninfected healthy individuals was used as a control. The MGH Institutional Review Board approved the study, and each subject provided written informed consent.

### Antibody purification

For functional studies, human antibodies were isolated from 100 µL of plasma. For passive transfer, IgG isolation was performed on 100 mL of plasma from a single elite controller or 100 mL of pooled plasma from five healthy controls using protein A followed by protein A/G Sepharose columns. Rhesus macaque antibodies were isolated from plasma using a Melon Gel IgG Purification Kit, as recommended by the manufacturer (Thermo Scientific). No differences were observed in antibody subclass distribution or function following melon gel or protein A/G purification. IgGs were also tested for their ability to interfere or block SHIV/HIV infection *in vitro*, and no effect was observed.

### Animals

Eleven Mamu-A*01-, B*17-, and B*08-negative adult Indian-origin rhesus monkeys were housed at the New England Primate Research Center, as recommended by the Guide for the Care and Use of Laboratory Animals. All studies were approved by the Institutional Animal Care and Use Committee (IACUC) of Harvard Medical School. Standard nutritious monkey diets were provided to each animal three times a day by skilled veterinary staff, in accordance with NEPRC standard operating procedures for veterinary care. No food or water deprivation occurred during the study. Animals were housed one per cage because socialized housing of animals under these circumstances increases the risk of transmission of the infectious agents, which could invalidate the study. Sources of enrichment included manipulation devices, foraging opportunities, food items, structural and environmental enhancements, and positive human interaction. Enrichment devices were rotated on a weekly basis and included toys, mirrors, radios, TV/VCRs, foraging boards, and a variety of complex foraging devices. To alleviate suffering, animals were fasted for 12 hours prior to any procedure and were given anesthesia, Ketamine HCL (10–20 mg/kg IM) for blood draws and colorectal biopsies. Following sample collections, the animals were monitored by the veterinary staff until recovered. Animals were humanely euthanized 3 months post antibody infusion by barbiturate overdose following anesthesia, in accordance with IACUC and HVMA guidelines.

### Challenge experiment

Five rhesus macaques received intravenous transfer (IV) of 50 mg/kg of the antibodies isolated from the elite controller, five rhesus macaques received IV transfer of 50 mg/kg of antibodies isolated from the healthy controls and one rhesus macaque received 25 mg/kg of the neutralizing monoclonal antibody b12 one day prior to intrarectal challenge with SHIV-SF162P3. SHIV-SF162P3 was generated in rhesus PBMC and was administered intrarectally at an intermediate dose (7.3 log RNA copies/mL or 340 TCID50) designed to infect the majority but not all of the animals.

### Sample collection

Plasma samples were collected on days 1, 3, 7, 14, 21, 28, 56 and 84 post-challenge and stored at −80°C. The plasma viral load was determined using the commercially available branched DNA (bDNA) kit (Siemens).

### ADCVI assay

Peripheral blood mononuclear cells (PBMCs) were prepared from the whole blood of healthy control rhesus macaques using density gradient centrifugation and Ficoll-paque PLUS (GE Healthcare). CD4^+^ T cells were obtained using a CD8 Depletion Kit (Stem Cell Technology) and cultured (5×10^6^/mL) for 3 days in RPMI 1640 supplemented with 10% fetal bovine serum, 2 mM L-glutamine, 100 µg/mL penicillin, 100 U/mL streptomycin, 10 mM HEPES, 50 U/mL IL-2 and 5 µg/mL PHA to activate the cells. On day 3, NK cells were isolated from the same donor rhesus macaque using a CD3 Depletion Kit (Stem Cell Technology). On the same day, the activated CD4^+^ T cells were infected with SHIV-SF162P3 at an MOI of 0.01 for 4 hours at 37°C. CD4^+^ T cells and NK cells were plated in 96-well round-bottom plates at an effector:target ratio of 10∶1 in the presence or absence of 50 µg/mL of antibody from the elite controller or the negative donor. Infected CD4^+^ T cells incubated with 50 µg/mL of antibody in the absence of effector cells served as a control to measure the capacity of each HIV-specific antibody to neutralize the virus and was complementary to the TZMBL assay, also aimed at measuring antibody neutralizing activity [27]. After 4 days of culture, the supernatant was collected, and viral replication was quantified using a p27 ELISA (Advanced Bioscience Laboratories). The inhibition of viral replication was calculated as follows: % Inhibition  =  (100-[100*{p27 concentration in wells containing effectors, targets and antibody}/{p27 concentration in wells containing targets and antibody}]).

In addition, we measured the ability of 50 ug/mL of human plasma purified antibodies to recruit human NK cells and suppress JRCSF or SF162 viral replication in activated human CD4 T cells. Briefly, peripheral blood mononuclear cells (PBMC) were prepared from the whole blood of HIV-negative donors using Ficoll Hypaque density gradient centrifugation. CD4s were obtained following 3 days of PBMCs culture at 5×10^6^/mL in RPMI 1640 supplemented with 10% fetal bovine serum, 2 mM L-glutamine, 100 ug/mL penicillin, 100 U/mL streptomycin, Hepes and 50 U/mL of IL-2 in the presence of 0.3 ug/mL of anti-CD3/8 antibodies. On day 3, autologous NK cells were isolated from the same donor using a Rosette Sep enrichment kit (Stem cell). On the same day, CD4 cells were infected with JRCSF or SF162 at a multiplicity of infection (MOI) of 0.01 for 4 hours at 37 degrees. Both CD4s and NK cells were washed and plated in 96 well round bottomed plates at an effector:target ratio of 10∶1 in the presence or absence of 50 ug/mL of antibody from Elite Controllers or HIV negative individuals. Infected CD4 cells incubated with 50 ug/mL of antibody in the absence of effector cells served as a control to measure the capacity of each antibody to neutralize the virus. After 4 days of culture, the supernatant was collected and the viral replication was quantified by p24 ELISA (Perkin Elmer). Inhibition of viral replication was calculated as follows: % Inhibition  =  [100-((p24 concentration in wells containing effector-target-antibody)/(p24 concentration in wells containing effector-antibody)*100)]. NK and CD4 T cells were prepared from a minimum of two different healthy individuals to perform the ADCVI assay.

### HIV-specific antibody titer quantification

ELISA plates (Nunc) were coated overnight at 4°C with 80 µL of PBS containing 250 ng/mL recombinant YU-2 gp120, SF162 gp120 or SHIVgp140SF162p3 (Immune technology). The wells were washed six times with PBS containing 0.05% Tween 20, blocked with 100 µL of PBS containing 5% BSA at room temperature for 2 hours and washed again. Serial dilutions of antibodies starting at 1 mg/mL in 45 µL were then added to each well, and the plates were incubated at room temperature for 2 hours. A pool of immunoglobulins from HIV-positive individuals (AIDS Reagent Program, Division of AIDS, NIAID, NIH; Catalog #3957, HIV-IG from NABI and NHLBI) was used as a positive control. After six washes, 100 µL of horseradish peroxidase–conjugated anti-human IgG (1∶500, diluted in PBS; BD Pharmingen) was added to each well, and the plate was incubated for 1 hour at room temperature, washed and developed by the addition of 50 µL of O-phenyl-enediamine diluted in 11 mL PBS. The reaction was stopped, and the optical densities at 492 and 605 nm were read on a Tecan ELISA reader. The data were analyzed in Prism, and the EC50 was calculated for each sample as follows: Y = Min+[(Max−Min)/(1+10^LogEC50-X^)] where Y is the observed value, Min is the lowest observed value, Max is the highest observed value, X is the value when the response is halfway between Max and Min and LogEC50 is the logarithm of the EC50.

### TZM-bl cell–based neutralization assay

**Figure 1:**
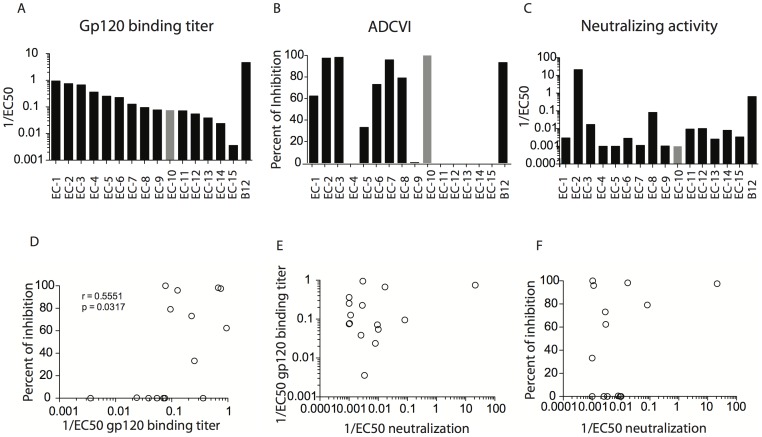
Polyclonal antibodies from naturally infected donors have a wide range of non-neutralizing anti-viral activities. Antibodies purified from the plasma of 15 different elite controllers (labeled EC-1 to EC15) were tested for their ability to bind to gp120 by ELISA, shown as the inverse of EC50 (A), to mediate ADCVI, measured as a percent of inhibition of viral replication (B) and to neutralize HIV virus (JRCSF), measured as an inverse of EC50 (C). The correlation plots show the relationship between gp120-specific antibody titers from elite controllers and ADCVI activity (D), or virus neutralization (E) as well as the association between the neutralization and ADCVI activity of antibodies (F). The elite controller from which the purified antibodies had the greatest innate immune recruiting properties in the absence of neutralization, EC10, was selected for passive transfer into macaques and is highlighted in gray. A minimum of 2 separate experiments was assessed to select the EC antibodies that had the best activity.

The neutralizing capacity of antibodies purified from the plasma of elite controllers was assessed using a modified TZM-bl assay [4], [28]. Briefly, TZM-bl cells (1×10^4^) were plated in 96-well plates, cultured overnight, and neutralization assays were performed using 10 time dilutions of antibody, starting at a concentration of 1 mg/mL. The diluted antibodies were mixed with 0.005 MOIs of a fully replicating Tier 1A (SF162), Tier 2 (JRCSF), or SHIV-SF162P3 virus, after which the antibodies and virus were transferred onto TZM-bl cells. The controls included cells cultured in the absence of virus, in the absence of antibodies (negative control) or in the presence of 5 µg/mL of the b12 or b6 monoclonal antibodies (positive controls). After a 24-hour incubation at 37°C, the cells were analyzed for luciferase expression. The data were analyzed using non-linear regression (curve fit) using Prism, and the percent neutralization obtained was calculated after subtraction of the background as follows: 100 * (1-[{RLU in the presence of both virus and antibody}/{RLU in the absence of antibody}]). Any antibodies in which we observed neutralization below 50% at 50 µg/mL were considered non-neutralizing. The values reported in Figure 1C correspond to the concentration of antibody necessary to achieve at least 50% neutralization of both the Tier 1A and Tier 2 viruses.

### Antibody-dependent cellular phagocytosis (ADCP)

Biotinylated antigen was incubated with 1 µm yellow-green fluorescent neutravidin beads (Invitrogen) overnight. The beads were then washed and resuspended at a final dilution of 1∶100 in PBS-BSA. Antibodies and 9×10^5^ antigen-labeled beads were mixed in a round bottom 96-well plate, and the plate was incubated for 2 hours. THP-1 cells (2×10^4^ cells) were then added to each well in a final volume of 200 µL, and the plate was incubated overnight under standard tissue culture conditions. The next day, half the culture volume was removed and replaced with 100 µL of 4% paraformaldehyde before the plates were analyzed on a BD LSR II equipped with an HTS plate reader. For analysis, the samples were gated on live cells, and the proportion of THP-1 cells phagocytosing beads was determined. The data represents the results of two separate experiments for which a phagocytic score was calculated as follows: (% bead positive x MFI bead positive [29]).

### Rapid fluorometric antibody-dependent cellular cytotoxicity (RFADCC)

The rapid fluorometric ADCC (RFADCC) assay was performed as previously described [30]. Briefly, 1×10^6^ CEM.NKr cells (Dr. Peter Cresswell through the AIDS Reagent Program, Division of AIDS, NIAID, NIH) were pulsed with 6 µg of recombinant gp120 (YU-2) for 1 hour and then washed twice in cold R10. Uncoated CEM.NKr cells were used as a negative control. Both the coated and uncoated target cells were stained with 1.5 µM PKH26 (Sigma) and 100 nM 5-(and-6)-carboxyfluorescein diacetate, succinimidyl ester (CFSE). After staining, the cells were resuspended at a concentration of 4×10^5^ cells/mL, and 2×10^4^ cells were added to each well. Macaque antibodies were added (500 µg/mL), and the plates were incubated for 20 minutes at 37°C and 5% CO_2_ to allow the binding of the antibodies to the target cell. NK cells (2×10^5^) isolated from healthy rhesus macaques using a customized CD3 Depletion Kit (Stem Cell Technology) were added to each well (a final effector:target ratio 10∶1). The plates were then incubated for 4 hours at 37°C, after which the cells were fixed in 2% paraformaldehyde. ADCC activity was determined using flow cytometry, on a minimum of two separate donors and was based on the loss of CFSE on PKH^+^ CEM.NKr cells. The data were analyzed using FlowJo software, and the percentage of CFSE loss within the PKH^+^ CEM.NKr population was determined.

### Surface Plasmon Resonance (SPR) assay

The ability of antibodies to bind to FcRs was determined using surface plasmon resonance (Biacore). The CM5 sensor chip was activated by injecting 25 µL of a mixture of an equal mixture of NHS (0.1 M in water) and EDC (0.1 M in water) at 7 µL/min. Next, 4.5 µg/mL of FcR diluted in 10 mM acetate buffer (pH 4.5) was injected over the chip surface at 7 µL/min for 10 min to coat. Unreacted sites were subsequently deactivated by injecting 25 µL of 1 M ethanolamine (pH 8.5) at 7 µL/min. 125 ug/mL of antibody were then run over the chip at a flow rate of 25 µL/min. The affinity (RU), on-rate (K_a_), off-rate (K_f_) and overall binding affinity (K_d_) was compared among the different groups.

### Antibody dependent complement deposition assay

The ability of antibodies to bind and activate the complement was determined by the C3b deposition on gp120-pulsed target cells. Briefly, 1×10^6^ CEM.NKr cells were pulsed with 6 µg of recombinant gp120 YU-2 or SF162 for 1 hour at room temperature and then washed twice in cold R10. Uncoated CEM.NKr cells were used as a negative control. Plasma from healthy individuals was collected and used as a source of complement for the assay. 25 ug of both the EC antibodies selected for the in vivo challenge and the antibodies purified from each 11 monkeys were added to 10^5^ CEM.NKr cells in the presence of plasma diluted 1∶10 in veronal buffer supplemented with 0.1% gelatin for 20 minutes at 37 degrees. Cells were washed and stained with a FITC-conjugated C3b antibody for 15 minutes, washed and fixed in 4% PFA. HIVIG was used a positive control for this assay and heat inactivated plasma as well as antibodies from healthy individuals were both used as negative controls. The data are the results of two separate experiments.

### Statistical analyses

All statistical analyses were performed using GrapPad Prism 5.0 software. Spearman rank correlation was used. Comparative analyses of the viremia between both groups were performed using an unpaired two-tailed t-test with 95% confidence intervals. *P*-values were two-sided and not adjusted for multiple testing. *P*-values less than 0.05 were considered statistically significant.

## Results

### Characterization of polyclonal ADCVI-inducing non-NAbs for passive transfer experiments

While previous passive transfer experiments in which non-neutralizing, ADCC-inducing antibodies were transferred failed to protect macaques from infection [25], these experiments were performed in neonatal rhesus macaques in which immature NK cells with poor functionality or insufficient antibodies may have contributed to an incapacity to form the immune complexes necessary to stimulate the low affinity Fc receptors. Therefore, we aimed to test whether a polyclonal pool of human non-neutralizing antibodies could provide protection from infection. Previous reports have shown that ADCC-inducing antibodies are significantly enriched in subjects who spontaneously control HIV infection in the absence of therapy, suggesting that these polyclonal antibodies may contribute to viral containment *in vivo* [12], [13], [17]. Since functional non-neutralizing antibodies from normally progressing macaques have led to a reduction in viremia after infection both by passive transfer and following vaccination [21]–[25], we performed a passive transfer of highly functional, non-neutralizing antibodies from selected spontaneous controllers exhibiting robust innate immune–recruiting activity to assess the potential impact of polyclonal non-neutralizing antibodies on a stringent SHIV-SF162P3 challenge. Thus, to identify a polyclonal pool of antibodies with robust innate immune–recruiting activity in the absence of neutralizing activity, we tested antibodies from 15 elite controllers. HIV-specific binding antibodies were observed in all but one subject (Figure 1A). By contrast, the observed ADCVI activity was heterogeneous, with robust functional activity detected in only half of the tested subjects (Figure 1B). Neutralizing antibody activity, measured in a TZM-bl assay, was very low in nearly all subjects tested, except for EC-2 (Figure 1C). Finally, ADCVI activity was only weakly correlated with the total gp120-specific antibody titer (Figure 1D) and neutralizing antibody activity showed no relationship with gp120-specific titers (Figure 1E). Furthermore, ADCVI and neutralizing antibody activity were unrelated to one another (Figure 1F). Based on these data, the subject with the lowest neutralizing antibody activity and the highest ADCVI activity (EC10; Figure 1A–C grey bars) was selected for large-scale antibody collection. Bulk antibodies were purified from a 100 mL plasma sample from EC10 using protein A/G columns. In addition, antibodies from a pool of healthy, HIV-negative individuals, were purified in parallel.

**Figure 2:**
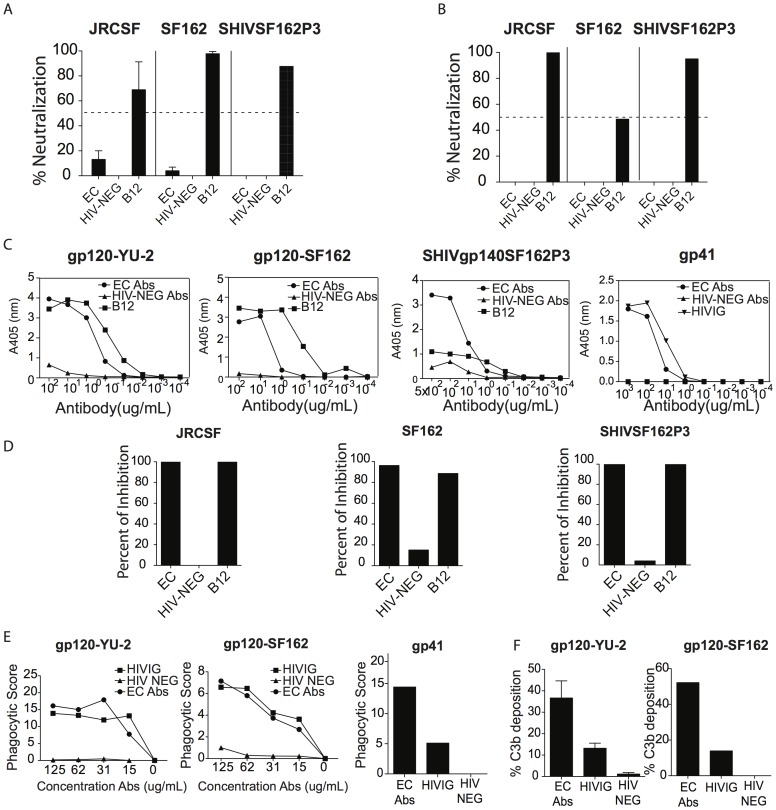
Antibodies purified from the elite controller selected for the *in vivo* study display a wide range of innate immune recruiting properties. Large quantities of antibodies were purified from the plasma of EC10 and assessed for their neutralizing capacity in a TZM-bl assay (A) and in a primary CD4 T cell assay (B). IgG HIV-binding titer against YU-2, SF162 gp120s, SHIVgp140 SF162p3 and gp41 was assessed (C) along with ADCVI activity (D) against a tier 2 (JRCSF), a tier 1A (SF162) and the challenge virus (SHIV-SF162P3). Purified b12 and a pool of antibodies purified from HIV-negative individuals were used as positive and negative controls, respectively. HIVIG was used as a positive control for testing gp41-binding titer. Antibodies purified from the plasma of EC10 were also evaluated for their ability to induce ADCP against a tier 2(JRCSF), a tier 1A (SF162) virus as well as against gp41 (E) and for their ability to induce complement activation as measured by C3b deposition on YU-2 or SF162 gp120 pulsed CEM cell line using HIVIG and a pool of antibodies purified from healthy individuals as positive and negative controls, respectively (F). A minimum of 2 separate experiments was performed to confirm the innate immune recruiting properties of the antibodies from the selected EC.

Purified IgG antibodies were reassessed for functional activity to confirm the previously defined functional profile. As expected, the purified antibodies from EC10 did not neutralize JRCSF, SF162 or SHIV-SF162P3 virus when measured using two distinct methods: a TZM-bl assay (Figure 2A) as well as a primary CD4 T cell culture (Figure 2B) that captures the neutralizing activity of antibodies targeting native envelope proteins, including gp41, for which the TZM-bl assay has been previously shown to be unreliable [3]. Following purification, the antibodies isolated from EC10 continued to exhibit potent gp120-specific titers against YU-2, SF162, gp140 specific antibody titer against SHIV-gp140-SF162P3 (Figure 2C; EC10, EC50 = 3.4 µg/mL; b12, EC50 = 0.4 µg/mL against YU-2) and potent gp41-specific antibody titers (Figure 2C; EC50 = 1.45 µg/mL). Of note, p24-specific antibodies were also detected in EC10 (data not shown). Similarly, purified antibodies purified from the HIV-negative donors continued to exhibit no gp120-reactivity or neutralizing activity (Figure 2A-C). In addition, both the purified EC10 antibodies and the positive control b12 exhibited potent ADCVI activity against the three tested viruses, whereas this activity was absent from the antibodies purified from the HIV-negative individuals (Figure 2D). Because antibodies are known to display a wide variety of Fc-mediating antiviral activities, we also evaluated their ability to mediate phagocytosis and to recruit complement. Notably, we observed that the purified EC10 antibodies were able to induce potent gp120 (using YU-2 and SF162 proteins)- and gp41- specific ADCP response (Figure 2E) and activate complement deposition on YU-2 or SF162 pulsed target cell lines (Figure 2F) whereas both activities were absent in antibodies purified from the healthy individuals suggesting that using different gp120 proteins, similar functionality were detected. Thus, the isolated EC10 antibodies maintained their potent Fc-mediated antiviral activity following large-scale purification.

### ADCVI-inducing non-NAbs do not protect from infection

The purified antibodies from EC10 or HIV-negative controls were administered intravenously at 50 mg/kg, approximating the upper limit of HIV-specific monoclonal antibody passive transfer and at roughly less than half of the dose of b12 required to achieve sterilizing protection [8], [31]-[33] and allowing for the delivery of polyclonal antibodies from a single donor to 5 animals. One animal received 25 mg/kg of the monoclonal antibody b12 that was used as an internal control since the protection of b12 has been previously reported to mediate protection from infection *in vivo* [8]. All antibodies were delivered 1 day before a single intermediate dose intrarectal challenge with SHIV-SF162P3 virus; viral infection was monitored at days 1, 3, 7, 14, 28, 56 and 84 days post transfer, and samples were collected at the same time points.

**Figure 3:**
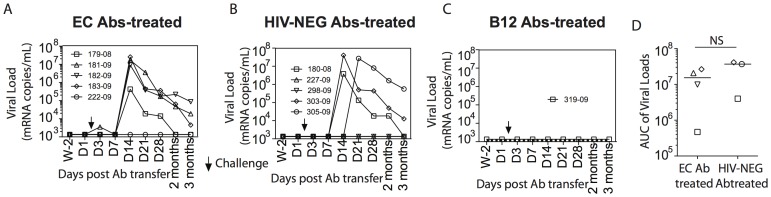
Kinetics of plasma viremia following SHIV-SF162P3 challenge. 50 mg/kg of antibodies purified from the selected elite controller (A) or from HIV-negative individuals (B) were passively transferred intravenously one day prior to the challenge with SHIV-SF162P3 in 5 rhesus macaques each. The plasma viremia was measured 14 days before and 1, 3, 7, 14, 21, 28, 56 and 84 days after antibody transfer. No viremia was detected in the animal that received 25 mg/kg of the b12 monoclonal antibodies used as a positive control. The area under the curve (AUC) of viral loads was measured in the 4 infected monkeys that received the EC antibodies and the 3 infected monkeys that received HIV-NEG antibodies (C). An unpaired two-tailed t-test with 95% confidence intervals was used to compare the AUC between both groups.

No difference was observed in the frequency of infected macaques among animals that received antibodies from EC10 (four of five infected) or the antibodies from HIV-negative individuals (three of five infected) (Figure 3A and B, respectively). In contrast, the single, proof of concept, control animal that received the b12 antibody remained uninfected with no detectable viremia at month 3 post infection confirming that the challenge virus can be blocked by a well-characterized monoclonal antibody (Figure 3C).

Additionally, no differences were observed in peak viremia (EC Ab-treated, mean 1.041e+007 copies/mL; HIV-NEG Ab-treated, mean 8.831e+006 copies/mL) or viral set point (EC Ab-treated, mean 144808 copies/mL; HIV-NEG Ab-treated, mean 1.693e+006 copies/mL) following infection (Figure 3). Moreover, the area under the curve (AUC) analysis of viral loads in the monkeys receiving the EC Abs (median AUC = 1.53e+007) compared to the infected monkeys that received the HIV-NEG antibodies (AUC = 3.96e+007) were not statistically different (Figure 3C). Thus, the passive transfer of polyclonal non-neutralizing antibodies from an elite controller with potent innate immune-recruiting activity was not able to protect rhesus macaques from or after infection following a stringent rectal SHIV-SF162P3 challenge.

### gp120-specific antibody titers and function do not correlate with post-infection viral control

**Figure 4:**
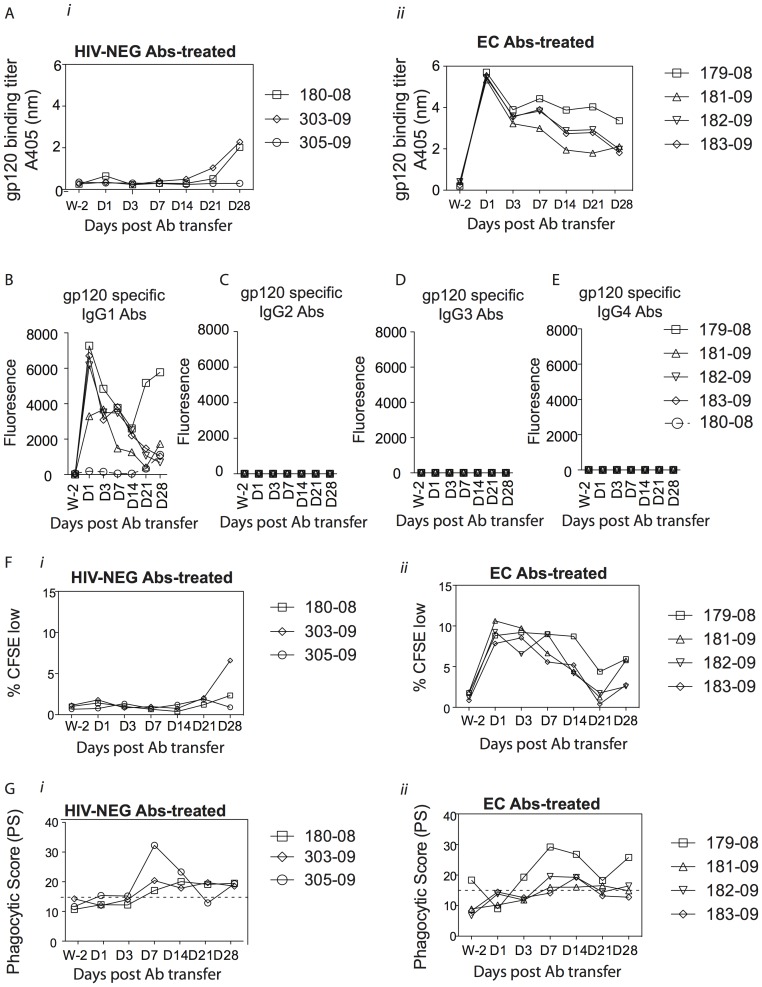
Kinetics of gp120-specific antibody titers and innate immune recruiting properties of antibodies following SHIV-SF162P3 challenge. ELISA was used to test the level of gp120-specific antibodies in the plasma of infected rhesus macaques (A) that received purified antibodies from either the pool of HIV-negative individuals (*i*) or the elite controller (*ii*). Optical Density (OD) values are reported. The level of gp120 specific antibody subclass over time was measured by Luminex in the plasma of the 4 infected monkeys that received the EC antibodies as well as in one monkey that received the HIV-NEG antibodies and is reported as mean fluorescence intensity (B–E). Antibodies were purified from the rhesus monkeys that received the EC antibodies (*i*) or the HIV-NEG antibodies (*ii*) and tested for their ability to recruit NK cells to mediate ADCC (F) or to recruit monocytes to mediate phagocytosis (G) 2 weeks prior the antibody transfer and at day 1, 3, 7, 14, 21 and 28 post antibody transfer. NK cells isolated from 2 separate donors were used to assess ADCC and 2 separate experiments were performed to measure the phagocytic activity.

Previous data suggest that the passive transfer of non-neutralizing, ADCC-inducing antibodies leads to post-infection viral control [25], [34]. To investigate parameters that may have accounted for the lack of protection, we next evaluated the dynamics of passively transferred polyclonal, non-neutralizing antibodies. First, we measured gp120-specific antibody titers in the plasma of all 7 infected monkeys. As expected, no gp120-specific antibodies were detected in the plasma of macaques that received antibodies from HIV-negative controls at early time points; however, two of the control Ab–treated rhesus macaques mounted gp120-specific antibody responses at Day 21 (Figure 4A *i*). By contrast, gp120-specific antibodies were detectable at Day 1 post-transfer in animals receiving the elite controller antibodies, and these antibody levels slowly decayed over the study period. Some variation was observed in the plasma antibody levels as early as Day 3, suggesting differential antibody-clearance among the macaques (Figure 4A *ii*). Moreover, nearly all of the circulating HIV-specific antibodies in the macaques were of the IgG1 subclass, suggesting that other antibody subclasses, which were detectable prior to transfer, may have been cleared rapidly after transfer (Figure 4B–E). Mucosal gp120-specific antibodies were not detectable in the macaques at Day 7 post-transfer, suggesting that either the antibodies penetrated the mucosa poorly, thereby providing only limited protection at the site of inoculation, or that the antibodies were cleared rapidly following transfer (data not shown). Thus, in spite of the detection of high gp120-specific antibody titers at early time points in all monkeys, no antibodies were detected at mucosal barriers and only IgG1 antibodies persisted in the blood.

**Figure 5:**
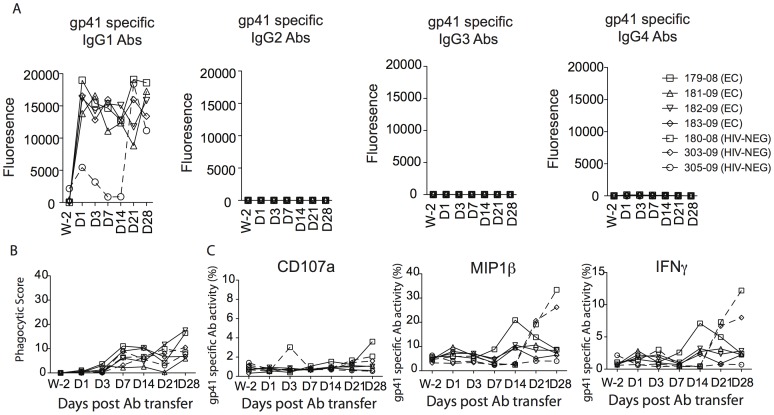
Kinetics of gp41-specific antibody titers and contribution of Fc mediated gp41 specific antibody responses following SHIV-SF162P3 challenge. The level of gp41 specific antibody subclass over time was measured by Luminex in the plasma of the 4 infected monkeys that received the EC antibodies as well as in one monkey that received the HIV-NEG antibodies and is reported as mean fluorescence intensity (A). Antibodies were purified from the rhesus monkeys that received the EC antibodies or the HIV-NEG antibodies and tested for their ability to recruit monocytes to mediate phagocytosis (B) or to activate NK cell measured as a release of CD107a, MIP1β and IFNγ (C) 2 weeks prior the antibody transfer and at day 1, 3, 7, 14, 21 and 28 post antibody transfer.

Next we evaluated the functional properties of transferred Abs, including gp120-specific ADCC, ADCP and complement deposition as well as gp41-specific ADCC and ADCP activities. As expected, no ADCC or ADCP activity was detected in the control animals (Figure 4F *i* and 4G*i*, respectively). However, while both gp120-specific ADCC and ADCP activities were observed in all four macaques that were infected following the transfer of EC antibodies (Figure 4F *ii* and 4G*ii*, respectively), these Fc mediated functions correlated only weakly with viral control post infection. Additionally, while complement activity was detected in the EC Ab preparation prior to infusion, no complement deposition was detected in any of the animals following antibody infusion suggesting that antibody mediated complement deposition were rapidly cleared following the passive transfer (data not shown). Similarly, while gp41-specific ADCP activity was readily detectable prior to infusion, and gp41-specific Ab titers were clearly detectable post-infection (Figure 5A), no gp41-specific ADCP activity or antibody dependent NK cell activation was detectable following passive transfer in the monkeys that received the EC antibodies (Figure 5B and 5C, respectively). These data suggest the potential preferential clearance of gp41-specific functional antibodies and all complement activating antibodies following passive transfer.

Overall, these data show that the passive transfer of a polyclonal pool of non-neutralizing antibodies with potent ADCVI activity did not protect from infection following a stringent SHIV-SF162P3 challenge. Antibody transfer dynamics revealed limited delivery of antibodies to the rectal mucosa and the selective loss of antibody-subpopulations following passive transfer potentially limiting the functional properties of the transferred polyclonal antibodies. These data raise the possibility that either non-neutralizing antibodies may not provide protection from or after infection or that other Fc mediated antiviral functions, alone or in combination, may have a greater impact on viral control. Thus, larger passive transfer experiments in which higher concentrations of antibodies are infused, or delivered specifically to the sites of challenge, may help definitively prove the protective role of non-neutralizing functional antibodies.

## Discussion

Recent results from the correlates analysis of the RV144 vaccine trial [35] pointed to a protective role of non-neutralizing antibodies in the control of HIV infection. Specifically, in the absence of vaccine-elicited IgA responses, several features of the antibody functional profile, including ADCC activity, were correlated with a reduced risk of infection. However, over the past decade, attempts to examine the protective role of non-neutralizing antibodies in the non-human primate model [21], [22], [24] have failed to mechanistically link antibodies that lack neutralizing activity to protection from infection. However, these negative results may be the result of several inherent difficulties associated with study design using non-neutralizing antibodies, including 1) the use of single monoclonal antibodies [31], [32], [36] [37] or pooled monoclonals [33], [38]–[42] [43], which target a limited number of epitopes and therefore induce poor immune complex formation, and 2) the use of NHP polyclonal sera with ADCC activity, which has not been associated with the durable antiviral activity observed in long-term non-progressors. To circumvent these problems, we performed a passive transfer experiment in rhesus macaques using polyclonal, ADCC-inducing antibodies isolated from an elite controller that exhibited non-neutralizing control of infection. No sterilizing protection from infection was observed following a rectal SHIV-SF162P3 challenge. Subsequent *ex vivo* analysis of transferred antibodies demonstrated unexpected antibody clearance post-transfer resulting in minimal antibody delivery to the rectal mucosa, selective loss of all non-IgG1 antibody subclasses in the blood, selective depletion of gp41-specific ADCP antibodies, and elimination of all complement activating antibodies. Such alterations in antibody subpopulations could profoundly alter *in vivo* functionality and therefore protective activity. However, despite this alteration, weak, but insignificant associations, were observed between gp120-specific titers and Fc-receptor binding characteristics with post-infection viral control (data not shown).

While the passive transfer of broadly neutralizing antibodies has been successful in mediating sterilizing protection in rhesus macaques, vaccines that elicit these responses have proven elusive. More recently, vaccine strategies that induce non-neutralizing functional antibodies have shown only limited success in preventing infection [21]–[24]. However, unlike neutralizing antibodies that simply block a limited number of viral epitopes on the surface of a virus, non-neutralizing antibodies must form avid immune complexes that are able to recruit the low-affinity receptors or innate immune proteins necessary for their function. Therefore, it is not surprising that the transfer of a polyclonal pool of non-neutralizing antibodies with the capacity to form these immune complexes may be required for protection. However, while the passive transfer of polyclonal sera, such as IVIG, is usually 8 times higher than the dose selected here (400 mg/kg), the bioactive dose of sialated anti-inflammatory antibodies or pathogen-specific antibodies are only a minute fraction of the total transferred antibodies [44]. Notably, it has been previously shown that HIV-specific antibodies constitute approximately 2% of the serum antibody pool [44], suggesting that the total fraction of HIV-specific antibody transferred in this study may have only approximated 1 mg/kg, at roughly less than half of the dose of b12 required to achieve sterilizing protection, potentially accounting for reduced protective efficacy. While these levels may be sufficient to block infection by some of the newer potent broadly neutralizing antibodies [45], significantly higher levels are likely required to generate enough immune complexes able to eliminate incoming virus. Recently, passive immunization in macaques with a polyclonal pool of anti SHIV IgG conclusively showed the critical nature of the transferred antibody dose [46]. Of note, the study demonstrated that passive transfer of 25 mg/kg of antibodies increased acquisition in a complement dependent manner, while partial protection was observed at 625 mg/kg, where HIV-specific antibodies still only constituted a transfer of 12.5 mg/kg. This data offers compelling evidence that protection can be achieved with non-neutralizing antibodies, and that perhaps the transfer or induction of similar doses of highly functional antibodies may offer even greater protection from infection. Thus future passive transfer with higher levels of elite controller antibodies, at ten-fold higher levels, may be required to achieve protection from infection, but may show enhanced protection from infection in the absence of neutralization.

Because most HIV transmissions occur across mucosal surfaces, the development of a successful, protective vaccine will likely require the presence of HIV-specific antibodies that harbor potent functional properties at the mucosal barrier. In the current study, transferred EC antibodies were not detected in the rectal mucosa at Day 7, potentially contributing to the lack of observed protection. Thus, it is plausible that either only low levels of the systemically administered antibodies reached the rectal mucosa and/or that the antibodies that reached the mucosa were cleared more rapidly in tissues compared to the blood. Therefore, future non-neutralizing antibody transfer studies may aim to deliver antibodies directly at the site of inoculation to define the critical humoral parameters involved in antiviral control.

While no protective activity of the passively transferred antibodies was detected, ancillary analyzes revealed that gp120 specific, but not gp41 specific, ADCC and ADCP were detectable in all 4 infected monkeys that received the EC Abs following infection, 2 functions that have been previously shown to control HIV replication in both humans and rhesus macaques [7], [12], [17], [47]. Of note, a blip in the ADCP activity was observed in one of the monkeys that received the HIV-negative antibodies following challenge, which was repeatedly shown to be non-HIV-specific. Because the HIV envelope protein is highly glycosylated, it is plausible that in this animal, natural antibodies may have emerged following infection, mimicking some of the autoimmune-like antibody responses documented in subjects that elicit broadly neutralizing antibody response against HIV [48]. Additionally, while Fc mediated antiviral properties of antibodies are tightly linked to their ability to bind to Fc-receptors and that we observed a weak but not statistically significant correlation between Fc-receptor binding and post infection viral control (data not shown), future efforts aimed at selecting sera with potent Fc-receptor binding, as a surrogate of robust Fc mediated activity, may help conclusively define the role for non-neutralizing antibodies in protection from infection.

Thus overall, no sterilizing protection was observed following the intravenous passive transfer of a low dose of non-neutralizing ADCVI-mediating antibodies using a rectal SHIV-SF162P3 challenge. While previous studies have pointed toward a crucial role for ADCC activity in viral control in both humans and rhesus monkeys, future experiments using an increased concentration of functional antibodies, both peripherally and at the site of challenge, may contribute to lowered viremia following infection. Despite a lack of protection, this study provides critical insights for the design of future animals studies aimed at conclusively defining the antiviral effect of polyclonal non-neutralizing HIV-specific antibodies.
